# Supplementary material for: Identification of photoperiod-induced specific miRNAs in the adrenal glands of Sunite sheep (Ovis aries)
Source: Front Vet Sci. 2022 Jul 22;9:888207. doi: 10.3389/fvets.2022.888207 (PMC9354845; doi:10.3389/fvets.2022.888207)
Supplement: Supplementary file 1 [file Data_Sheet_1.ZIP › Supplementary file/miRDeep2 file.zip!/miRDeep2 file/Novel_Structure/SP42A1/Novel_411.pdf]

Provisional ID	:	NC_019464.2_11227
Score total	:	0.7
Score for star read(s)	:	-1.3
Score for read counts	:	0
Score for mfe	:	0.4
Score for randfold	:	1.6
Score for cons. seed	:	
Total read count	:	28
Mature read count	:	28
Loop read count	:	0
Star read count	:	0

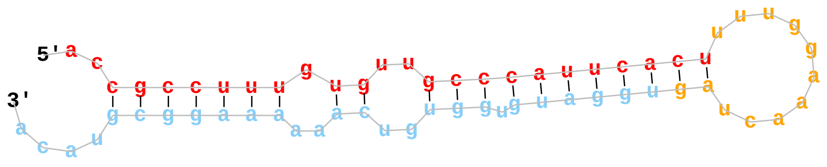

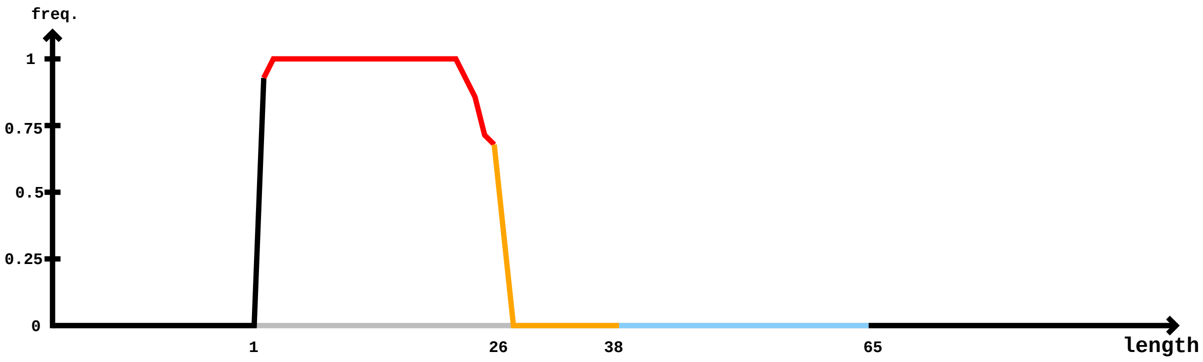

Mature **Star**

```
5'- auuccuuaugcgguuuucuuaccgccuuuguguugcccauucacuuuuggaaacuaguggauguggugucaaaaaaggcguacacaaaacacuuucgcagcuuuuuuuccugccc  -3'   exp
    ............(((((.....(((((((.((..(((((((((((..........)))))))).)))..))..))))))).....)))))......((((.........))))..   reads   mm   sample
    ....................accgccuuuguguugcccauu..........................................................................   2       0    seq
    ....................accgccuuuguguugcccauuc.........................................................................   2       0    seq
    ....................accgccuuuguguugcccauuca........................................................................   4       0    seq
    ....................accgccuuuguguugcccauucac.......................................................................   1       0    seq
    ....................accgccuuuguguugcccauucacu......................................................................   15      0    seq
    ....................accgccuuuguguugcccauucacuu.....................................................................   2       0    seq
    .....................ccgccuuuguguugcccauucacu......................................................................   1       0    seq
    .....................ccgccuuuguguugcccauucacuu.....................................................................   1       0    seq
```
